# Acute malnutrition recovery rates improve with COVID-19 adapted nutrition treatment protocols in South Sudan: a mixed methods study

**DOI:** 10.1186/s40795-023-00696-y

**Published:** 2023-03-11

**Authors:** Emily Lyles, Sandra Banks, Maya Ramaswamy, Sule Ismail, Eva Leidman, Shannon Doocy

**Affiliations:** 1 Department of International Health, Johns Hopkins Bloomberg School of Public Health, 615 N Wolfe St, Baltimore, MD 21205 USA; 2 US Centers for Disease Control and Prevention, Atlanta, GA USA; 3 US Centers for Disease Control and Prevention, Juba, South Sudan; Integral Global Consulting, Tucker, GA, USA

**Keywords:** COVID-19, Acute malnutrition, Community management of acute malnutrition, South Sudan

## Abstract

**Background:**

Globally, emergency nutrition program adaptations were implemented as part of COVID-19 mitigation strategies, but the implications of the adoption of all protocol changes at scale in the context of deteriorating food security are not yet well characterized. With ongoing conflict, widespread floods, and declining food security, the secondary impacts of COVID-19 on child survival in South Sudan is of great concern. In light of this, the present study aimed to characterize the impact of COVID-19 on nutrition programming in South Sudan.

**Methods:**

A mixed methods approach including a desk review and secondary analysis of facility-level program data was used to analyze trends in program indicators over time and compare two 15-month periods prior to the onset of COVID-19 (January 2019 – March 2020; “pre-COVID period”) and after the start of the pandemic (April 2020 – June 2021; “COVID” period) in South Sudan.

**Results:**

The median number of reporting Community Management of Acute Malnutrition sites increased from 1167 pre-COVID to 1189 during COVID. Admission trends followed historic seasonal patterns in South Sudan; however, compared to pre-COVID, declines were seen during COVID in total admissions (− 8.2%) and median monthly admissions (− 21.8%) for severe acute malnutrition. For moderate acute malnutrition, total admissions increased slightly during COVID (1.1%) while median monthly admissions declined (− 6.7%). Median monthly recovery rates improved for severe (92.0% pre-COVID to 95.7% during COVID) and moderate acute malnutrition (91.5 to 94.3%) with improvements also seen in all states. At the national level, rates also decreased for default (− 2.4% for severe, − 1.7% for moderate acute malnutrition) and non-recovery (− 0.9% for severe, − 1.1% for moderate acute malnutrition), with mortality rates remaining constant at 0.05–0.15%.

**Conclusions:**

Within the context of the ongoing COVID-19 pandemic in South Sudan, improved recovery, default, and non-responder rates were observed following adoption of changes to nutrition protocols. Policymakers in South Sudan and other resource-constrained settings should consider if simplified nutrition treatment protocols adopted during COVID-19 improved performance and should be maintained in lieu of reverting to standard treatment protocols.

**Supplementary Information:**

The online version contains supplementary material available at 10.1186/s40795-023-00696-y.

## Background

At the onset of the COVID-19 pandemic, more than half of the population of South Sudan (SSD) faced crisis or worse levels of food insecurity, and several areas were at risk of famine [1]. Acute food insecurity was estimated to be worst in part of Greater Upper Nile (Jonglei, Unity, Upper Nile states) and Bahr el Ghazal (Lakes, Warrap, and Northern Bahr el Ghazal states) as of February 2020 [2]. The food security situation deteriorated in 2020 due to violence, widespread displacement, historic flooding, a weak economy, and COVID-19 pandemic related supply chain disruptions. Livelihoods in rural parts of the country were negatively impacted by movement restrictions, and food prices surged, constraining both food availability and access [1]. Given this context, the United Nations (UN) projected in January 2021 that more than 313,000 children would experience severe acute malnutrition (SAM), and more than 1 million children would experience moderate acute malnutrition (MAM) during the year [3].

Community Management of Acute Malnutrition (CMAM) programs are designed to help children recover from acute malnutrition. The vast majority of children with acute malnutrition are managed on an outpatient basis and recover within a several month period [4]. In South Sudan, CMAM programs are implemented by nongovernmental organizations (NGOs) at health facilities throughout the country. Children visit facilities on a weekly or bi-weekly basis for health and weight checks and to receive nutrient and calorie rich ready-to-use foods (RUF).

To avoid health facilities becoming epicenters of transmission, UNICEF, the Global Nutrition Cluster (GNC), and the Global Technical Alliance Mechanism for Nutrition (GTAM) released interim guidance for Management of Child Wasting in the Context of COVID-19 on March 27, 2020 [5]. In addition to guidance to improve infection prevention and control protocols, there were recommended revisions to: (1) admission criteria (such that only children with low mid-upper arm circumference (MUAC) or oedema, but not low weight-for-height (WHZ) would be eligible), (2) dosing of therapeutic foods to be fixed amounts rather than based on child weight, (3) reduce the frequency of follow-up visits, and (4) rely on community-based platforms as feasible [5, 6].

The South Sudan Ministry of Health released ‘Guidelines for Nutrition Service Delivery in the Context of COVID-19’ on March 31, 2020, adopting all adaptations recommended in global guidance [7]. The guidelines were later revised to change the frequency of follow-up from weekly to bi-weekly for the treatment of SAM and bi-weekly to monthly for the treatment of MAM and guidance released in April 2020 clarified that in the absence of weight measurements, children should be provided with two sachets of ready-to-use therapeutic food (RUTF) per day for SAM and one sachet of ready-to-use supplementary food (RUSF) for MAM regardless of weight [8]. Updated guidance in August 2021 added the need to meet MUAC-based discharged criteria for two consecutive visits prior to discharging a child as cured [9]. The frequency of follow-up was returned to weekly for SAM and biweekly for MAM in August 2021; other mitigation measures remain in place [10].

The implication of changing context on CMAM admissions has large operational implications for the management of CMAM programs. If rising food insecurity is associated with an increase in acute malnutrition prevalence, this would drive an increase in CMAM admissions; however, protocol changes may have the opposite effect. Suspension of WHZ as an admission criterion excludes a subset of children otherwise eligible for treatment of acute malnutrition and suspension of mass screening activities could decrease program coverage. Following admission, protocol simplifications such as reduced visit frequency and simplified dosing could impact both recovery rates and length of stay, though this is not yet well characterized. Some studies have identified non-inferior outcomes when select simplifications were implemented [10, 11]. However, generalizability to the South Sudan context requires further investigation to understand the implications of simultaneous adoption of all protocol changes at scale in a non-research setting and in the context of deteriorating food security.

## Methods

A mixed methods approach consisting of a desk review and secondary analysis of facility-level program data was used. The analysis focused on trends over time and descriptive comparison of two 15-month periods: 1) January 2019 – March 2020 (“pre-COVID period”), and 2) April 2020 – June 2021 (“COVID” period).

### Desk review

A review of contextual factors (e.g., floods, conflict intensification) that may have impacted nutrition program performance concurrent to the impacts of the pandemic was conducted with a focus on information available from government, humanitarian NGOs, and United Nations sources. Searches were performed across relevant public databases and websites (e.g., Relief Web, Humanitarian Response, the South Sudan nutrition cluster, FEWS NET, and the EM-DAT Emergency Database). Several publicly available reports were identified prior to web searches and were incorporated into the review (e.g., humanitarian snapshots, situation updates, and UN and government response plans). Situational timelines of key factors impacting nutrition programming, including conflict, natural disasters, and climate were developed at national and state levels, with state-level timelines incorporating county-level detail when available.

### Quantitative secondary data analysis

Partners operating CMAM sites for treatment of SAM and MAM in South Sudan are requested to report monthly aggregate performance indicators by facility to the South Sudan Nutrition Cluster. For children 6–59 months of age, reported performance indicators include monthly number of children enrolled and exiting from each site, as well as exit outcomes (e.g., recovered, death) by sex and type of program of enrollment (i.e., outpatient therapeutic programs (OTP) for SAM and targeted supplementary feeding programs (TSFP) for MAM). These site-level data shared by the Nutrition Cluster, consisting of routine reporting from all CMAM sites in South Sudan (i.e., comprehensive country-wide data rather than a sample) from January 2019 through June 2021, were analyzed to evaluate changes in nutrition programming within the context of the COVID-19 pandemic [4, 12].

Indicators were analyzed separately for SAM and MAM and included: 1) SAM/MAM admissions: Number of children 6–59 months of age admitted to OTP and TSFP programs, respectively; and 2) SAM/MAM program exit outcomes: Number of children exiting from OTP/TSFP by outcome type (recovered, default, death, and non-response) as a proportion of all children existing in each program. These proportions (commonly referred to as recovery, defaulter, death, and non-response rates) are standard indicators of program performance. All indicators are presented both monthly and as monthly medians for each 15-month period (pre-COVID and COVID). Secondary analysis was conducted at national and state levels with a focus on overall trends between January 2019 and June 2021, as well as descriptive comparison of change between the pre-COVID and COVID periods across states; no statistical testing was performed. Data are exhaustive, reported from all facilities operating CMAM activities during the review period, not a sample; thus, all analysis was unweighted. No adjustments were made for sites with missing data for select months.

The study was reviewed and approved by the South Sudan Ministry of Health Ethics Committee and Institutional Review Board at Johns Hopkins Bloomberg School of Public Health. This activity was reviewed by the US Centers for Disease Control and Prevention (CDC) and was conducted consistent with applicable federal law and CDC policy.

## Results

The national analysis of CMAM program data included sites operated by 58 partners, including both national and international NGOs. A total of 1852 unique CMAM sites provided data for at least 1 month during the review period, including 120 operating only OTPs, 75 operating only TSFPs, and 1657 operating both. Of all sites, 257 (14%) reported data for all 30 months under review. The number of CMAM sites reporting ranged from 1082 to 1197 in the pre-COVID period and 1164–1223 during COVID with the median number of CMAM sites increasing from 1167 pre-COVID to 1189 during COVID. The monthly median number of CMAM sites reporting data increased in seven of the ten states, with only Upper Nile (− 24), Northern Bahr el Gazal (− 11), and Lakes (− 9) states reporting decreases (Additional file 1).

### Admissions

South Sudan experiences seasonal trends in nutrition program admissions, characterized by an increase in monthly admissions between the beginning of the year (January) and spring (April to June), and a decrease through the second half of the year. Prior to the onset of the pandemic, seasonal declines in SAM and MAM admissions were observed from May to December 2019, which were followed by enrollment increases in the first quarter of 2020. Following the onset of COVID-19, trends for both SAM and MAM admissions were similar, though there was less month-to-month variation in SAM admissions compared to the previous year.

**Fig. 1:**
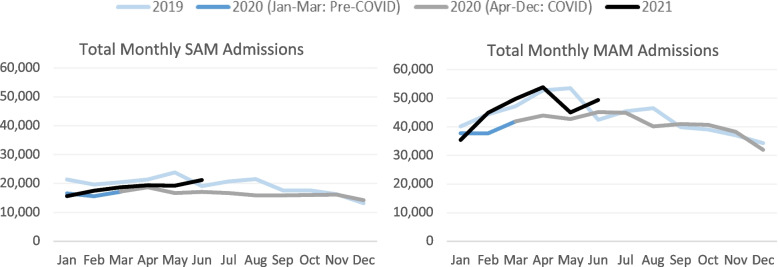
Trends in Monthly SAM and MAM Admissions Over Time

**Fig. 2:**
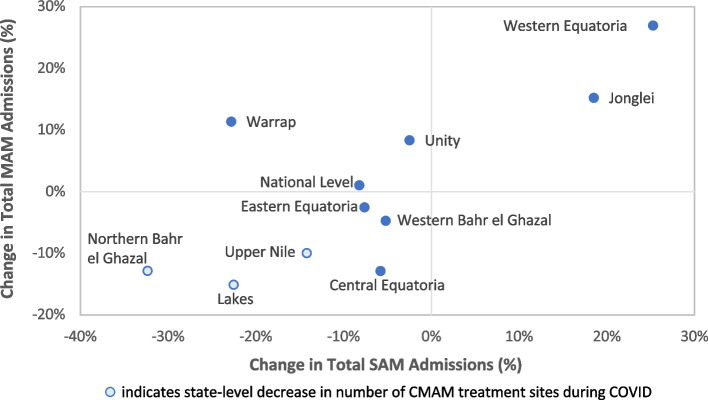
Change in Total SAM and MAM Admissions by State (percent change pre-COVID [January 2019-March 2020] to COVID [April 2020-June 2021] periods)

Aggregated by period, the total number of SAM admissions declined by 8.2% during COVID-19 (from 282,289 pre-COVID to 259,067 during COVID) despite the increase in the number of reporting sites. The total number of MAM admissions increased slightly by 1.1% (from 639,752 pre-COVID to 646,877 during COVID) (Fig. 1). Total admissions for SAM and MAM decreased during COVID in eight and six states, respectively. Western Equatoria and Jonglei were the only states with increases in total admission for both SAM and MAM (Fig. 2). In eight of ten states, the directional change of SAM and MAM admissions was the same; the exceptions were Warrap and Unity, which saw decreases in SAM admissions and increases in MAM admissions. Northern Bahr el Ghazal, Lakes, and Upper Nile had the largest declines in admissions and were also the only states to have fewer CMAM sites reporting during COVID.

Given relatively large fluctuations in the absolute number of admissions in select sites, median monthly admissions were also reviewed. Nationally, median monthly admissions decreased by 21.8% for SAM and 6.7% for MAM between periods (Table 1). At the state level, changes in median monthly SAM admissions ranged from − 38.3% (Upper Nile) to 25.4% (Western Equatoria); for these states, changes in median monthly MAM admissions were similar, ranging from − 34.9% (Upper Nile) to 24.9% (Jonglei).

**Table 1:** Change in median monthly SAM and MAM admissions during COVID-19

	Median Monthly SAM Admissions			Median Monthly MAM Admissions		
	Pre-COVID	COVID	% Change^a^	Pre-COVID	COVID	% Change^a^
National	**21,036**	**16,457**	**−21.8%**	**45,984**	**42,901**	**−6.7%**
Central Equatoria	860	708	− 17.6%	2696	2060	−23.6%
Eastern Equatoria	1937	1479	−23.6%	4354	3491	−19.8%
Jonglei	3100	4128	33.2%	6772	8459	24.9%
Lakes	2182	1432	−34.4%	4796	3559	−25.8%
Northern Bahr el Ghazal	2992	1953	−34.7%	6357	5715	−10.1%
Unity	3066	2662	−13.2%	5714	6032	5.6%
Upper Nile	1643	1014	−38.3%	3856	2511	−34.9%
Warrap	2528	1772	− 29.9%	5952	6219	4.5%
Western Bahr el Ghazal	1550	1136	− 26.7%	2954	2533	−14.3%
Western Equatoria	890	1116	25.4%	2535	2657	4.8%

^a^Comparison of 15-month periods before (Jan 2019 to Mar 2020) and after (April 2020 to June 2021) COVID

The ratio of children admitted for treatment of MAM to those admitted for SAM treatment pre-COVID was 2.3 compared to 2.5 during COVID, with state-level ranges of 2.0–3.1 pre-COVID and 2.0–4.0 during COVID. The largest shift in MAM:SAM admissions ratio was in Warrap, which was the only state with opposing admissions trends: a 22.8% decrease in SAM admissions in parallel with an 11.8% increase in MAM admissions.

### Recovery rates and other treatment outcomes

**Fig. 3:**
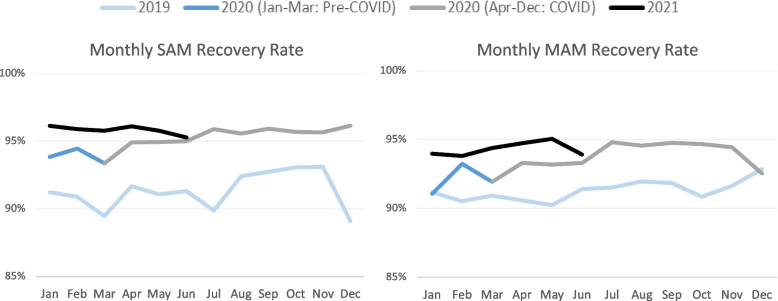
Trends in Monthly SAM and MAM Recovery Rates Over Time

Monthly recovery rates of children with SAM from March 2020 onwards exceeded those of the corresponding month in the pre-COVID period. Monthly recovery rates for MAM increased slightly from March 2020 through late 2020 and remained consistently above recovery rates for the corresponding month of 2019 through November (Fig. 3). Monthly SAM recovery rates from January 2019 to February 2020 ranged from 89 to 94% compared to 93–96% from March 2020 forward, and median monthly recovery rates improved from 92.0% pre-COVID to 95.7% during the COVID period. For MAM, monthly recovery rates ranged from 91 to 93% from January 2019 to February 2020 and increased to 92–95% from March 2020 forward, with the median monthly recovery rate increasing from 91.5% pre-COVID to 94.3% during COVID.

All states saw improvements in median monthly recovery rates for both SAM and MAM during COVID (Table 2). The greatest improvements in median monthly SAM recovery rates were seen in Upper Nile (7.9%) and Central Equatoria (5.6%), which had the lowest pre-COVID recovery rates. The highest median monthly SAM recovery rates during COVID were in Western Bahr el Ghazal (98.2%) and Western Equatoria (97.9%). Median monthly MAM recovery rates increased the most between periods and at similar levels (4.0–4.6%) in Western Bahr el Ghazal, Eastern Equatoria, Upper Nile, and Northern Bahr el Ghazal. During COVID, the highest recovery rate was in Western Bahr el Ghazal (98.0%), and the lowest was in Northern Bahr el Gazal (88.6%). Unity State had the smallest increase in median monthly SAM and MAM recovery rates, though this was attributed to having the highest pre-COVID recovery rates (97.0% for SAM and 95.9% for MAM). With the exception of Aweil Centre County in Northern Bahr el Gazal, all counties had SAM and MAM recovery rates above the 85% treshold set out in the Sphere Standards [13]. With pre-COVID recovery rates of 72.6% for SAM and 61.8% for MAM, Aweil Centre was an outlier. Recovery rates for SAM and MAM increased by 0.5 and 7.4%, respectively, from the pre-COVID to COVID period in Aweil Centre, however, gains were insufficient to meet performance standards.

**Table 2:** Change in median monthly SAM and MAM recovery rates during COVID-19

	Median Monthly SAM Recovery Rate			Median Monthly MAM Recovery Rate		
	Pre-COVID	COVID	% Change^a^	Pre-COVID	COVID	% Change^a^
National	**92.0%**	**95.7%**	**3.9%**	**91.5%**	**94.3%**	**3.1%**
Central Equatoria	86.7%	91.5%	5.6%	88.1%	90.4%	2.6%
Eastern Equatoria	91.5%	95.4%	4.3%	90.8%	94.8%	4.4%
Jonglei	90.7%	94.2%	3.8%	92.8%	94.3%	1.6%
Lakes	93.8%	97.5%	3.9%	93.3%	96.3%	3.2%
Northern Bahr el Ghazal	91.4%	93.3%	2.0%	85.1%	88.6%	4.2%
Unity	97.0%	97.3%	0.3%	95.9%	96.3%	0.4%
Upper Nile	90.3%	97.4%	7.9%	92.7%	96.7%	4.3%
Warrap	92.0%	95.6%	3.9%	91.7%	95.4%	4.0%
Western Bahr el Ghazal	94.8%	98.2%	3.6%	93.7%	98.0%	4.6%
Western Equatoria	93.8%	97.9%	4.4%	92.7%	96.2%	3.8%

^a^ Comparison of 15-month periods before (Jan 2019 to Mar 2020) and after (April 2020 to June 2021) COVID

While recovery rates increased for both SAM and MAM, the total number of children recovered decreased by 8.3% for SAM with varied direction of change across states, and by 1.8% for MAM (Table 3). This change is primarily due to declines in CMAM program admissions. The greatest reduction in the number of SAM children recovered was in Northern Bahr el Ghazal, where there was a similar proportional decrease in median monthly SAM admissions (a common occurrence). The greatest increase in the number of MAM children recovered was in Warrap (17.3%), and the greatest decrease was in Central Equatoria (14.9%).

**Table 3:** Change in total counts of children by exit outcome during COVID-19^a^

	SAM / OTP Program Exit Counts				MAM / TSFP Program Exit Counts			
	Non-Recovered	Default	Death	Cured	Non-Recovered	Default	Death	Cured
**National**	**−43.1%**	**−58.9%**	**−35.2%**	**− 8.3%**	**−23.4%**	**− 40.0%**	**− 42.1%**	**1.8%**
Central Equatoria	−37.9%	− 39.1%	−63.3%	− 3.0%	−31.8%	−28.7%	136.8%	− 14.9%
Eastern Equatoria	−17.3%	−55.7%	− 48.8%	3.0%	−24.1%	− 44.0%	−51.0%	11.6%
Jonglei	−23.2%	−49.8%	2.4%	8.3%	−33.6%	−14.1%	10.7%	−8.1%
Lakes	−17.7%	−69.1%	−51.2%	−11.1%	23.7%	−58.2%	37.0%	−0.6%
Northern Bahr el Ghazal	−35.1%	−59.1%	− 78.0%	−31.2%	−25.1%	− 41.2%	−92.5%	−6.4%
Unity	− 26.5%	−46.9%	100.0%	−3.7%	23.0%	−12.2%	− 86.3%	3.6%
Upper Nile	−93.7%	−79.4%	−45.0%	−16.5%	−49.6%	−48.3%	−50.0%	−2.6%
Warrap	−50.9%	−66.5%	−35.8%	−21.2%	−20.0%	−49.7%	−36.3%	17.3%
Western Bahr el Ghazal	−61.9%	−67.9%	300.0%	10.6%	−56.4%	− 73.0%	716.7%	13.8%
Western Equatoria	−42.7%	−66.7%	−35.3%	14.0%	−24.8%	−62.3%	7.7%	−0.2%

^a^ Comparison of 15-month periods before (Jan 2019 to Mar 2020) and after (April 2020 to June 2021) COVID

**Fig. 4:**
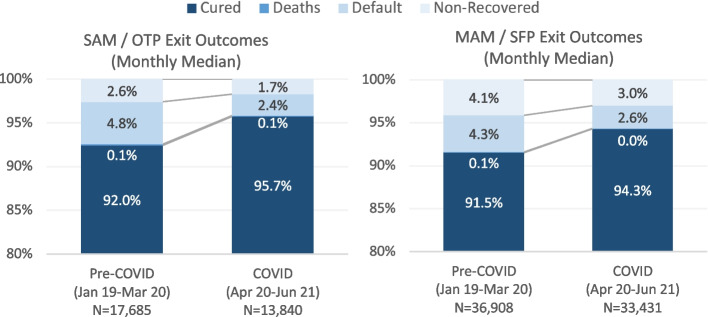
Change in Exit Outcomes for SAM and MAM During COVID-19

In addition to the proportion of children recovered (recovery rate), CMAM programs report the proportion of children that exit as non-recovered, defaulters, and deceased. The recovery, default, and non-recovered rates improved for both OTP and TSFP at the national level, with mortality rates remaining constant at 0.05–0.15%. The default rate declined from the pre-COVID to COVID period by 2.4% (4.8% pre-COVID vs. 2.4% during COVID) for SAM and 1.7% for MAM (4.3% pre-COVID vs. 2.6% during COVID). The non-recovered rate declined by 0.9% (2.6% pre-COVID vs. 1.7% during COVID) for SAM and 1.1% for MAM (4.1% pre-COVID vs. 3.0% during COVID) (Fig. 4). While median monthly mortality rates were stable for both SAM and MAM, the total number of SAM deaths fell by 35.2% (324 deaths pre-COVID vs. 210 during COVID), and the total number of MAM deaths decreased by 42.1% (324 deaths pre-COVID vs. 210 during COVID). The largest proportional increases in SAM deaths occurred in Western Bahr el Ghazal (300%) and Unity (100%), whereas the largest increases in the number of MAM deaths were in Western Bahr El Ghazal (717%) and Central Equatoria (137%).

## Discussion

This analysis aims to characterize changes in national-level nutrition program performance resulting from the COVID-19 pandemic and pandemic response, including modifications to nutrition treatment protocols and service delivery strategies. Analysis of programmatic data from all CMAM reporting sites in South Sudan found evidence of decreased admissions and improved program outcomes.

Total admissions and median monthly admissions for SAM both declined during the COVID period compared to pre-COVID; total MAM admissions increased slightly during COVID, while median monthly admissions declined. It is difficult to discern which contextual factors or protocol adaptations drove changes in admission given the regularity of flooding, displacement, intensification of conflict, and worsening food security concurrent to the pandemic’s emergence and implementation of CMAM protocol changes (Additional file 2); however, a similar analysis at the state level provides a more nuanced understanding of state- and county-level factors that most likely provoked changes in admissions [14].

Declining admissions in the context of extreme food insecurity, as observed during COVID-19, is notable. At a national level, food security was improving in late 2019; however, by October 2020, 6.4 million people were estimated to be acutely food insecure, and this figure remained above 6 million through 2021 [15]. Estimates of the number of children under five in need of treatment for acute malnutrition increased slightly from 1.2 million at the onset of the pandemic to 1.3 million 2021 [16, 17]. Of the counties where the nutrition situation was classified as critical from November 2020 to March 2021, nearly 80% were in Upper Nile, and 17% in Northern Bahr el Gazal, but both of these states saw decreases in SAM and MAM admissions, which is unexpected given crisis levels of food insecurity [1]. The number of CMAM admissions during the 15-month COVID period in this analysis equates to 66% of the projected 2021 acute malnutrition caseload nationally (83% for SAM and 60% for MAM), suggesting a potential gap between acute malnutrition treatment needs and recuperative services provided, particularly for MAM [3].

Changes in the CMAM treatment protocols adopted in South Sudan may contribute to the decline in admissions observed in the context of persistent food insecurity and other exacerbating contextual factors. Previous research has demonstrated that suspending the use of WHZ as an admission criterion would, on average, result in 25.8 and 49.6% of previously eligible SAM and MAM children, respectively, being excluded from treatment entirely, and 19.6% of children previously eligible for SAM treatment being eligible for MAM treatment [18]. Action Against Hunger, a primary provider of CMAM services in South Sudan, estimates that in 2019, 55% of children newly admitted to programs for management of SAM and 10% for MAM were admitted on the basis of WHZ alone and would be ineligible under revised protocols [with MUAC as the only admission criteria] [19].

This analysis provides evidence that nutrition program outcomes did not deteriorate, and potentially improved, during the COVID-19 period in South Sudan both nationally and in all states and, except for one county, were above the performance standard. The ecological analysis presented does not allow for a causal understanding of this finding, but does provide preliminary evidence supporting non-inferior outcomes of the revised protocols. Given concerns that default and non-response rates could potentially increase with reduced visit frequency, non-inferior program performance is a noteworthy finding. A recent study of outcomes following COVID-19 adaptations to acute malnutrition programs in Uganda, Ethiopia, and Somalia also found consistent recovery rates well within Sphere standards, further refuting the expectation of recovery declines with reduced follow-up visits [20].

Given the variations in protocol simplifications and combinations of adaptations implemented across contexts, and limited evidence on the impacts of widespread modifications during COVID-19, further research on how certain modifications (e.g., MUAC-only admissions, reduced visit frequency, and modified dosing) can impact outcomes that are designed to allow for individual-level adjustment for child characteristics is important.

The presented analysis is novel in that it presents trends over 30 months, a long enough period to capture pre-COVID seasonal trends, and includes data from all health facilities reporting they were operating CMAM programs in South Sudan during the review period. In contrast to previous analyses based on samples or sub-national datasets, this allows for a more comprehensive picture of trends in admissions and program outcomes following the onset of the pandemic.

The present analysis is nevertheless subject to several limitations. First, the data provided are aggregated facility-level counts; without individual level data, it was not possible to adjust for child-level demographic characteristics or nutritional status. Second, many sites reported no admissions for many months during the review period, but there is no documentation of whether this reflects site closures or gaps in reporting. Third, the analysis is observational in nature and not designed to understand the cause of the changes in admissions or program outcomes described*.* Finally, while the COVID period in our analysis begins in April 2020, there were numerous protocol revisions implemented at different times in different locations beyond this time.

## Conclusions

This analysis leveraged national program data to investigate changes in nutrition programming in South Sudan during the onset of the COVID-19 pandemic. The study builds on existing evidence, largely from pilots and research studies with smaller sample sizes, by analyzing national-level outcome data when protocol modifications were implemented at scale. Comparison of pre-pandemic and pandemic periods found a modest reduction in admissions during COVID that coincided with worsening food insecurity, conflict, displacement, and several significant floods. States with the largest admission declines also saw reductions in the number of treatment facilities during COVID, suggesting that coverage was a key issue. Given the situational complexity and large annual variations in admissions, it is unclear what changes in admissions, if any, are due to treatment protocol adaptations*.* Evidence to support nutrition treatment protocol simplifications, many of which were implemented at scale as COVID-19 mitigation strategies, is strong and suggests numerous benefits alongside similar treatment outcomes compared to standard protocols. Within the context of the ongoing COVID-19 pandemic in South Sudan, improved recovery, default, and non-responder rates were observed following adoption of changes to nutrition protocols. Policymakers in South Sudan and other resource-constrained settings should consider if simplified treatment protocols adopted during COVID-19 improved performance and should be maintained in lieu of reverting to standard treatment protocols.

## Supplementary Information

**Additional file 1.** Number of CMAM Sites by State during the Pre-COVID and COVID Periods.

**Additional file 2.** Timeline of Shocks and Drivers.

## Data Availability

The datasets analyzed during the current study are available from the South Sudan Nutrition Cluster’s online dashboard at https://public.tableau.com/views/South_Sudan_Dashboard2021V1/Dashboard1?:language=en-US&publish=yes&:display_count=n&:origin=viz_share_link

## Abbreviations

CDC: US Centers for Disease Control and Prevention
CMAM: Community Management of Acute Malnutrition
GNC: Global Nutrition Cluster
GTAM: Global Technical Alliance Mechanism for Nutrition
MUAC: Mid-upper arm circumference
MAM: Moderate acute malnutrition
NGOs: Nongovernmental organizations
OTP: Outpatient therapeutic programs
RUF: Ready-to-use foods
RUSF: Ready to use supplementary food
RUTF: Ready to use therapeutic food
SAM: Severe acute malnutrition
SSD: South Sudan
TSFP: Targeted supplementary feeding programs
WHZ: Weight-for-height
