# Supplementary material for: Acute malnutrition recovery rates improve with COVID-19 adapted nutrition treatment protocols in South Sudan: a mixed methods study
Source: BMC Nutr. 2023 Mar 11;9:46. doi: 10.1186/s40795-023-00696-y (PMC10008100; doi:10.1186/s40795-023-00696-y)
Supplement: Supplementary file 1 — Additional file 1. Number of CMAM Sites by State during the Pre-COVID and COVID Periods. [file 40795_2023_696_MOESM1_ESM.pdf]

Additional file 1: Number of CMAM Sites by State during the Pre-COVID and COVID Periods

	**Pre-COVID** Jan 2019- Mar 2020		**COVID** Apr 2020 - June 2021		Change in Median Number of Sites (COVID vs. pre-COVID)
	Median	Range	Median	Range	
Central Equatoria	67	52-75	76	72-86	9
Eastern Equatoria	155	147-160	163	158-164	8
Jonglei	166	153-175	185	157-206	19
Lakes	113	108-121	104	101-110	-9
Northern Bahr el Ghazal	135	118-138	124	119-129	-11
Unity	134	130-139	141	131-147	7
Upper Nile	150	113-167	126	117-138	-24
Warrap	111	90-125	119	114-124	8
Western Bahr el Ghazal	68	56-72	73	62-77	5
Western Equatoria	80	49-89	81	76-83	1
**Total**	**1167**	**1082-1197**	**1189**	**1164-1223**	**22**
