# Supplementary figures and images for: Acute malnutrition recovery rates improve with COVID-19 adapted nutrition treatment protocols in South Sudan: a mixed methods study
Source: BMC Nutr. 2023 Mar 11;9:46. doi: 10.1186/s40795-023-00696-y (PMC10008100; doi:10.1186/s40795-023-00696-y)

## Additional file 2: Timeline of Shocks and Drivers

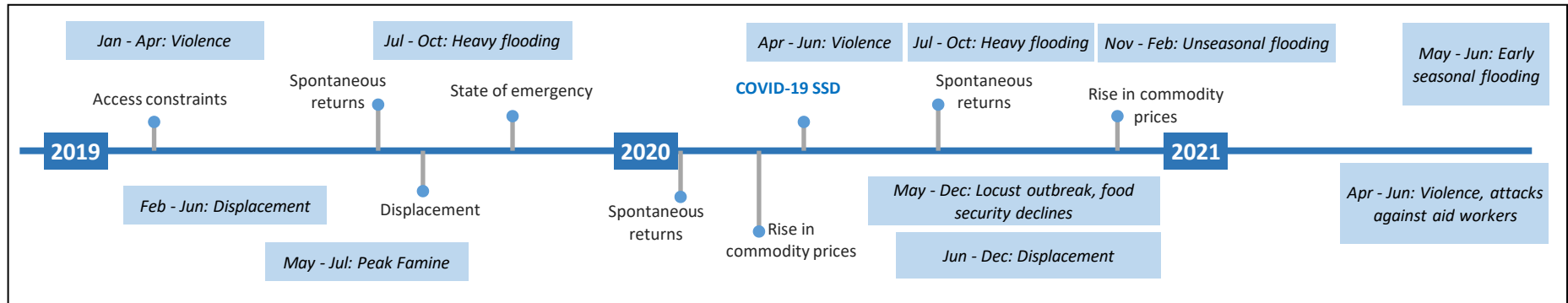

Supplement: Supplementary file 2 — Additional file 2. Timeline of Shocks and Drivers. [file 40795_2023_696_MOESM2_ESM.pdf]
